# The Light and Dark Sides of Student Engagement: Profiles and Their Association with Perceived Autonomy Support

**DOI:** 10.3390/bs12110408

**Published:** 2022-10-23

**Authors:** Dong Yang, Zhenyu Cai, Yu Tan, Chen Zhang, Mengti Li, Cheng Fei, Ronghuai Huang

**Affiliations:** 1 Smart Learning Institute, Beijing Normal University, Beijing 100018, China; 2 Yizhou Elementary School, Chengdu 610000, China

**Keywords:** school engagement, school burnout, autonomy support, latent profiles, demand-resource model

## Abstract

School engagement has assumed an important place in current developmental psychology and educational research due to its potential to address students’ low achievement, high dropout rates, and misbehavior. Although much has been written about the antecedents and outcomes of student engagement, literature on how students’ level of engagement differs in response to different teaching styles was missing on a large scale. Understanding the patterns and risks linked with student engagement provides opportunities for targeted intervention. This study explored primary school students’ engagement and burnout profiles and how different profiles interacted with perceived classroom teaching styles (i.e., autonomy-supportive & autonomy suppressive). Latent profile analysis resulted in four student engagement subgroups: moderately engaged, engaged, moderately burned out, and burned out. Students clustered into engagement groups were likely to report higher autonomy support from teachers. In contrast, burned-out groups were more likely to rate teachers’ teaching styles as suppressive (i.e., autonomy suppressive). Collectively, the study indicated that autonomy-supportive teaching behaviors are pivotal in understanding student engagement and school burnout. Thus, tailored teacher-focused intervention programs that enhance teachers’ awareness of autonomy-supportive teaching is important. The significance of the findings with the demand-resource model (in the education context) was discussed.

## 1. Introduction

The literature on student engagement has gained increasing popularity in education research, policy, and practice [1], due to its associations with desired academic and non-academic outcomes such as learning achievement, physical and psychological well-being [2]. Engagement was defined as the time and energy students devote to educational activities [3], positive emotions and learning strategies [4], desire, demand, and motivation to participate [5], or efforts in enriching students’ learning experiences and performance. Despite the significant variation in how engagement has been defined, there appears to be a consensus that engagement is a multifaceted construct that unites varying aspects of engagement, such as behavior, cognition, and emotion [6]. Salmela-Aro and Upadaya adapted the idea of work engagement to the school context, defining schoolwork engagement from perspectives of energy, dedication, and absorption, a definition corresponding to the behavioral, emotional, and cognitive aspects of student engagement [7]. Energy refers to high levels of energy and resilience while performing school-related tasks, and dedication refers to the experience of school-related work as meaningful and inspiring. At the same time, absorption depicts a pleasant state of total immersion in schoolwork or activities [8]. 

Meanwhile, current studies have also started with concerns about the negative aspects of engagement, especially disengagement [9,10]. As an antonym of engagement, disengagement is usually described as a fluid state that features coping mechanisms for dealing with distress, withdrawal, apathy, inertia, negative emotions, and psychological distress [11,12]. Disengaged students were resenting course demands, impatient, and bored with intellectual pursuits [13]. Compared with studies on engagement, the topic of disengagement is still undercover. From the perspective of developmental psychology, disengagement is seen as more than just a lack of engagement; it is a distinct psychological process with its own impact on students’ academic results, thus, to better understand and model engagement, both its positive and negative elements should be researched [14]. Moreover, the emotional state of disengagement was close to burnout and a psychological syndrome manifested as emotional exhaustion, cynicism, and a sense of inadequacy [15]. Exhaustion is characterized by fatigue, school-related ruminations, and sleep troubles. The manifestation of cynicism is an apathetic or distant attitude toward learning in general, a lack of pleasure in studying, and an inability to understand the significance of studying. While sense of inadequacy relates to a diminished sense of competence, achievement, and accomplishment. In general, school burnout can either be triggered by high perceived study demands, a cynical and detached attitude toward one’s studies, or feelings of Inadequacy [16,17], echoing emotional, cognitive, and behavioral aspects of school burnout [15]. The workplace burnout concept has lately been extended to school contexts, assuming that a school is where students work [8,18]. In accordance with this contemporary approach, we in this study define school burnout among elementary school pupils as a combination of exhaustion as a result of school demands, a cynical and detached attitude towards school, and feelings of inadequacy [19]. 

In a broader sense, school engagement and burnout can be defined as the quantity and quality of students’ involvement and disengagement in school or school activities [20]. Furthermore, engagement and burnout are multifaceted and feature dynamic ana d bilateral processes sensitive to the learning environment [6,21]. Engaged students are more likely to earn higher grades and better personal adjustment to school. At the same time, students who are burnt out are more likely to fail academically, drop out of school, and be exposed to various negative psychosocial consequences [22]. Moreover, scholars have agreed that school engagement and burnout are malleable states that can be shaped by school context [1,23]. Recent literature also emphasized the importance of the learning environment on students’ engagement and burnout. Many researchers have investigated the role of supportive socio-contextual factors in building students’ academic engagement [24]. One such aspect of support is students’ perceived teaching practice, which was generally conceptualized as a stable pattern in a teacher’s methods of instruction, classroom management, and interpersonal style with students [25]. Regarding style, a teacher can be supportive or suppressive [26]. Over the last two decades, ample evidence suggests how different teaching practices (e.g., motivational styles) could predict student engagement and burnout at school [27,28] (Assor et al., 2002; Jang et al., 2012). For example, both positive (e.g., providing choice, fostering understanding and interest) and negative indicators (e.g., intruding, suppressing criticism & independent opinions) of teacher motivational styles were studied across different subjects [25,27,29,30]. 

Research on student engagement profiles are not new. However, studies employing person-centered approaches (e.g., using latent profile analysis) are scarce [31]. Several works on engagement patterns in the K12 context can be identified, and quite commonly, typical profiles ranging from highly engaged to disengaged were identified [32,33,34]. More recently, studies also examined student engagement and burnout among adolescents. For instance, engagement and school burnout were used in person-centered analyses to identify profiles [35]. Five subgroups were clustered: high-engagement/low burnout, average-engagement/average-burnout, and low-engagement/high-burnout. Studies also found that factors most strongly associated with adolescents’ high-engagement/low-burnout profile were high levels of support from teachers and family and social-emotional skills [8,35,36]. In a longitudinal design, Wang and Peck profiled students’ engagement by gathering data from the students from ninth grade to one year after their expected high school graduation [37]. In general, highly engaged students showed the highest academic achievement, also had fewer depressive symptoms than the less engaged groups. Similarly, studies also found that youths in the highest engagement performed better academically and showed less delinquency and depression than students in decreasing profiles [38].

A large body of research on student engagement and burnout has been conducted in the context of college students [17,39,40] or recently on high school students [36,41,42]. However, still, little is known about engagement and burnout among young adolescents such as primary school students. For instance, in school burnout alone, literature on the context of primary school is missing on a large scale. Only two were located at the Chinese primary school level (one journal article & one dissertation), according to a recent systematic review [43]. The limited result also manifested in the literature on social support and student engagement [44]. In China, a typical East Asian country, students have been traditionally good at the tests, meanwhile stressed even at the primary school level due to fierce peer competition. Pressures have been identified as the essential sources of stress that Taiwanese adolescents experience [45], and also in mainland China [46,47]. Long-term pressure and burdens contribute to the development of burnout symptoms [48]. Therefore, identifying at-risk student groups in stress appraisals and burnout and providing interventions is crucial for their well-being and supporting successful academic pathways early on [49]. Students who are disengaged or ‘at risk’ of disengagement could facilitate targeted interventions to improve student outcomes [50]. This information will help develop and train academic practitioners to develop targeted interventions. Strategies could include formalized programs such as learning and social support services to more individualized educational guidance and counseling. 

To address the lack of literature in this respect, school burnout was used to depict the negative, dysfunctional part of emotional processes in school. At the same time, the concept of schoolwork engagement represents positive emotional processes in school. In particular, we in this study applied a person-oriented method to identify primary school students’ burnout and engagement profiles and investigate how these profiles differ in the critical teacher motivational style factors using the motivational and demotivational framework [27]. The main questions we asked were: (1) What kind of profiles can be identified regarding students’ school engagement and burnout? (2) How will those profiles differ in their association with perceived autonomy support from teachers? 

## 2. Methodology

### 2.1. Participants and Procedure

Participants were students recruited from three primary schools (10 classrooms) across a large Southwestern China city. The students and their parents were fully informed regarding the purpose of the study and procedure, and informed consent forms were collected. With the help of class teachers, we delivered 500 copies of questionnaires in pencil-and-paper format. The participants were given around 30 min to complete a set of questionnaires measuring school burnout, school engagement, perceived autonomy support, and various background variables. We get back 412 valid responses (Mage = 11.03 years, SDage = 0.89; 50% female), resulting in a response rate of 82.4%. According to previous studies, a sample size of at least 250 should work well with LPA [51,52]. The sample consisted of grade four (18%), grade five (26%), and mostly grade six (55%). The study protocol was endorsed by the ethics al review board of the researchers’ institute. 

### 2.2. Measures

School engagement was measured using a previously validated nine-item schoolwork engagement inventory [7], SEI measures three components of school engagement. (1) vigor/energy (three items., e.g., when studying, I feel strong and vigorous), (2) dedication (three items., e.g., I am enthusiastic about my studies/work), and (3) the state of absorption (three items., e.g., I feel happy when I am studying intensively). The replies were graded on a four-point scale (1 = very untrue of me; 4 = very true of me), and a total score (Cronbach’s = 0.93) was computed to measure the student’s overall school participation.

School burnout was accessed using School Burnout Inventory (SBI) [15]. Three components of school burnout experience: exhaustion at school (four items, e.g., I feel overwhelmed by my schoolwork); cynicism towards the meaning of school (three items, e.g., I’m continually wondering whether studying has any purpose), and sense of inadequacy (two items, e.g., I often have feelings of Inadequacy when studying) were measured. Responses were rated on a four-point scale (1 = very untrue of me; 6 = very true of me). This three-factor SBI has shown good reliability according to previous studies [53] (For validity and reliability, see reference 19). The Cronbach’s α reliabilities were computed separately for each dimension, α = 0.80, 0.83, and 0.82, respectively.

To measure the autonomy-supportive teaching practice, we adopted questionnaires from validated previous studies [27], in which they measured autonomy-enhancing and suppressing teacher behaviors using a six-component questionnaire: (1) *providing choice* (e.g., when I am doing something that interests me, teacher gives me enough time to finish it); (2) *fostering understanding and interest* (e.g., teacher talks about the connection between what we study in school and what happens in real life); (3) *allowing criticism and encouraging independent thinking*. (e.g., the teacher listens to my opinions and ideas); (4) *suppressing criticism & independent opinions*. (e.g., teacher is willing to listen opinions that fit her opinion); (5) *forcing meaningless and uninteresting activities* (e.g., teacher forces me to read boring things (books, stories or instructions) (6) *intruding* (e.g., teacher is strict about me doing everything in her way. Three items of each component were used (sum of 1, 2, 3 for *autonomy support*, while the sum of 4, 5, and 6 for *autonomy suppression*) for analysis. In addition, we did a confirmative factor analysis (CFA) on the two-factor variable, the result indicated that the two-factor CFA model fitted the data well (χ2 = 625.38, df = 134, *p* < 0.001, CFI = 0.96, SRMR = 0.06, RMSEA = 0.07, 90% CI for RMESA [0.05, 0.09]). Previous studies have shown good validity and reliability on this two-factor/dimension autonomy-supportive teaching styles [54,55]. Students’ answers were rated with a four-point scale (1 = strongly disagree; 6 = strongly agree). Cronbach’s α was formed separately for each dimension, and the value is primarily good at 0.78, 0.82, 0.80, 0.85, 0.90, and 0.81. For correlations between variables and descriptive statistics for all included variables, see Table 1 below.

### 2.3. Analysis Strategy

For the statistical analysis of the data, we applied a multistep approach. We initialed data analysis by computing the correlations between variables and descriptive statistics for all included variables. Next, latent profile analysis was performed to identify categorical latent classes of individuals using: the sum score of schoolwork engagement and three components of school burnout (i.e., exhaustion, cynicism, Inadequacy) as indicators [56]. LPA is useful when data contains continuous variables. There are individual differences among participants. These differences arise logically and can be investigated through patterns [57]. We choose those indicators as such structure of SEI and SBI has been used and supported in previous studies [36,58]. Then, we tested a succession of models with increasing numbers of profiles to find the best model of latent profiles. LPA model fit was compared using the statistical criteria included in the Mplus statistical modeling application: Akaike information criterion (AIC), Bayesian information criterion (BIC), Sample-size Adjusted BIC (SABIC), Lo-Mendell-Rubin likelihood ratio test (LMR) or Vuong-Lo-Mendell-Rubin likelihood ratio test (VLMR), Adjusted LMR (ALMR), bootstrapped likelihood ratio test (BLRT), and entropy value. Those are also the commonly used selection method [51]. Smaller AIC and BIC values suggest a better fit between the model and the data [59] or a higher likelihood of replication, while higher entropy values indicate better differentiation between latent profile groups [60]. The LMR/ALMR compares a k-1 profile model (H0) against a k-profile model; hence, a low *p*-value indicates that the model with one fewer profile should be rejected. In terms of entropy, a higher value, for example, values > 0.80 would suggest that the latent classes are highly discriminating [59,61,62].

As listed above, there are various indices for selecting an appropriate LPA solution, while it is common to rely on multiple criteria to finally determine a best-fit model [55,59,63]. Moreover, previous studies also suggested theories and related content considerations [64,65], which could further benefit the interpretation of those latent profiles extracted. Regarding multiple criteria for model selection, a recent review found most of the studies applied the BIC value as one of the model fit matters, while over 70% referred to the SABIC value, both of which were proved to perform well in detecting the collect number of classes with considerable distance between classes [52,63]. In addition, nearly 60% of past research used ALMR, which is more robust and may avoid overestimating profile enumeration than the other indices [65]. The size of profiles should not be too small [63]. For example, 1% or 3% could be a rule of thumb. Hence, to ensure the goodness of fit and the model parsimony, we took the following steps to determine the number of profiles: 1. Exclude those solutions that fail to meet the 0.8 thresholds of the entropy value; 2. Check the ALMR test results with *p* values exceeding 0.05, and consider those k-1 solutions; 3. Double check the possible solutions with their BIC value and the size of the smallest profile; 4. Decide among the remaining solutions based on theoretical deduction or practical coherence. 

## 3. Results

Table 2 lists the fit indices for the compared mixed model solutions that vary from one to five profiles. Based on engagement, exhaustion, cynicism and feelings of Inadequacy. Since the five-profile solution showed a nonsignificant result for the ALMR test and the entropy value started to decline, we considered the four-profile solution with the highest entropy value of 0.956 and an ALMR *p* value less than 0.01 over the three-profile solution. Next, although the BIC and SABIC values continued to reduce as the number of profiles increased, the dropping magnitude kept narrowing. Thus, the four-profile solution was probably reasonable compared to others.

**Figure 1:**
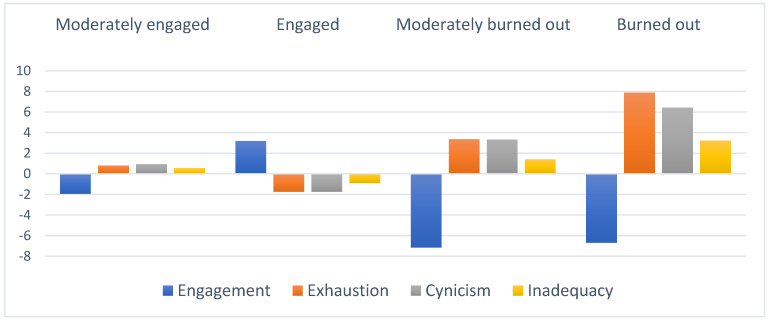
Standardized means on the clustering variables by profiles.

Moreover, the proportion of the smallest profile, 4%, met the required minimum size that should be at least 3% of the total sample size. Therefore, the four-group solution was chosen as the final latent group solution due to its good fit and was theoretically meaningful. Figure 1 depicts the final four-group solution, whereas Table 3 describes the unstandardized final solution.

In the first latent group (36%; *n* = 150), we labeled it the moderately engaged student group as it features a slightly above-average level of burnout symptoms (exhaustion, cynicism, feelings of Inadequacy) and a slightly below average level of engagement. The second latent group (50%; *n* = 207) was characterized by the lowest level of burnout symptoms in all aspects and a high level of engagement. Thus, it was named the *engaged* group. The third and fourth latent groups consisted of relatively small numbers of students: The third group, *moderately burned out* (9%; 36), features an above-average level of burnout symptoms and low level of engagement, while the fourth group (4%; 15) was characterized by highest levels of burnout symptoms and yet low level of engagement, it was therefore labeled the *burned-out* group.

To investigate the role of perceived autonomy support in predicting the latent groups, variables (autonomy support & autonomy suppress) were added to the final model as covariates. In terms of the size and interpretation of the latent groups, the resultant group memberships remained identical to those in the final LPA model after including these variables. According to the results, students in the classes with supportive teachers were likely to be engaged (or moderately engaged) in school. Meanwhile, the students who perceived teachers as suppressing teaching behaviors were likely to have burned-out symptoms (see Table 4).

## 4. Discussion

School is a crucial developmental environment for teenagers [66]. This study is one of the earliest to investigate the profiles of school engagement and burnout among primary school students to the best of our knowledge. Using a person-oriented approach, this study explored the latent profiles of study-related burnout symptoms (e.g., exhaustion, cynicism, and feelings of Inadequacy) and engagement among primary school students. In addition, it investigated the unique role of perceived autonomy support in predicting latent profile memberships. Four clusters that feature moderately or highly engaged and burned out were identified.

Interestingly, most students (86%) were clustered into either the moderately engaged or engaged groups, as they reported moderate to high engagement and average or below average levels at all study-related burnout symptoms. In comprise with recent studies, this is a relatively small portion. For example, a survey by Salmela-Aro and Katja Upadyaya found that 19% of the high school students belonged to the burned-out cluster, but much similar to the high-burnout (5.5%) of lower secondary schools in a recent study [35]. As predicted, teachers’ autonomy support was a critical predictor of the latent profiles. Students who depicted teacher’s motivational styles as autonomy supportive were more like to belong to the engagement profiles (i.e., moderately engaged or engaged). Meanwhile, those rated teachers featuring suppressive autonomy styles were more likely to fit into the burned-out clusters, indicating a clear linkage between perceived motivational styles and student engagement [25,27]. 

Moreover, our study identified a relatively low percentage (around 14%) of burnout profiles. This is a bit low percentage compared with other studies. For example, a study in France found high burnout symptoms among 34% of sixth-graders and 28% of fourth-graders [67]. In China, new government policy to lighten the schoolwork and homework burden of students and (financial) burden on parents [68] (In Chinese: 双减). This policy was issued based on the current situation that many Chinese students were stressed and exhausted by a tremendous amount of schoolwork and homework [45,46]. Hence, a longitudinal design would be interesting to investigate students’ engagement and burnout during the “double reduction” process.

Generally, people have a fundamental psychological need to be autonomous, competent, and connected to others [69]. Therefore, satisfying these basic needs (i.e., autonomy) promotes people’s motivation (i.e., participating in a task with a higher level of engagement), while thwarting the needs may make individuals feel stressed or burned out. For this reason, teacher educators need to monitor teachers’ teaching styles closely and hold meetings regularly to exchange good instructional practices that have the potential to maximize student engagement and minimize school-related burnout symptoms. Importantly, teachers can play a crucial role in promoting student engagement, since autonomy-supportive teachers might maximize student engagement via (a) nurturing inner motivational resources, (b) relying on noncontrolling informational language, and (c) acknowledging the students’ perspective and feelings [25]. When initiating learning activities, they might involve students’ inner motivational resources. By adopting their point of view and identifying and nurturing the students’ preferences and demands, teachers could set meaningful learning goals and present students with engaging, enriching activities related to their daily lives. Communicate noncontrolling and informatively, and acknowledge students’ opinions when bad sentiments and behavioral problems arise [70,71].

The context of this study can also refer to the demands-resources model, which was drawn from the original job demands-resources (JD-R) model of occupational stress [72,73]. From the perspective of learning, it divides the characteristics of (learning/school) environments into two genres: demands and resources [74,75]. Demands involve physical and psychological effort and, as a result, are associated with physiological and psychological consequences (i.e., academic burnout). Resources refer to all the elements (be it physical or psychological) that support the achievement of learning objectives in the learning process [73]. A recent study has interpreted study-related stress as a demand perceived by students in their school tasks and regarded a high self-efficacy as a resource for improving their engagement at school [76].

Similarly, the autonomy-supportive teaching behavior of this study can be interpreted as a school resource that has the potential to maximize student engagement. At the same time, insufficient autonomy support (i.e., being indifferent) or even autonomy suppressing could be regarded as a threat to engagement that leads to disengagement or burnout. Moreover, a previous study has proven that burnout is positively associated with depression [76]. This means to combat school burnout may further contribute to preventing depressive symptoms. 

## 5. Limitations & Future Directions

Limitations existed in almost every study; this study is not an exception. First, this study used questionnaire responses from students. Despite most salient aspects of the environment could only be understood by measuring how students perceive them [77], a better result can be obtained from other perspectives. For instance, inviting trained observers to rate teachers’ instructional styles and students’ engagement (i.e., behavioral engagement) and combine this with their subjective feelings of engagement and burnout. 

Moreover, based on a longitudinal design, research has found study demands were associated with school burnout a year later, while study resources were related to schoolwork engagement [75]. In addition to school demands and resources, *personal resources* (which depict the interactions between person and environment) have recently been added to the demands-resources model. Personal resources are crucial as they facilitate students’ goal achievement in the face of hardship [78]. Therefore, it may be of value to see how adolescents’ perceptions of school engagement and burnout changed during the “double reduction” policy, as well as how multiple personal resources (e.g., self-efficacy, academic buoyancy, grit) buffer the negative impact of burnout on engagement and well-being. 

Limitations also existed in the scope of this study. First, we found that students who rated teachers teaching styles as autonomy supportive are more likely to be engaged. However, readers should remember that student engagement (and burnout) are complex, fluid processes that feature a dynamic system of social and psychological constructs and synergistic processes [79]. Therefore, future studies should explore such topics in-depth by involving more social and psychological factors such as social support, grit, or academic buoyancy. Moreover, recently the work demands-resources model has been applied to the educational context, and there is already ample evidence that study demands and study resources were related to school burnout and schoolwork engagement separately [75]. Future studies can extend this work by adding more psychological factors into this model, such as support from others (i.e., social support), and examine its effectiveness in explaining the complex interaction between school/social offerings and the dynamic process of personal development.

## 6. Conclusions

Students in academic contexts face various study demands and resources, which may manifest in their degree of engagement and burnout at school and their general well-being [66]. We, in this study, explored students’ engagement and burnout profiles among primary school students and how different profiles interacted with perceived classroom teaching styles (autonomy-supportive vs. autonomy suppressive) from teachers. To date, this is one of the earliest attempts to investigate such relations from a primary school level. We successfully identified four clusters (i.e., moderately engaged, engaged, moderately burned out, and burned out) based on students’ reported engagement and burnout symptoms. In addition, we found that students of engagement profiles were more likely to report a higher level of satisfaction with autonomy support from teachers. At the same time, burned-out groups were more likely to rate teachers’ teaching styles as suppressive (i.e., autonomy suppressive). Taken together, this result may indicate the importance of instructional styles on students’ school engagement and its role as a buffer against academic adversity (such as burnout). This is an area that deserves in-depth exploration in follow-up studies. 

## Figures and Tables

**Table 1:** Pearson’s correlations among all variables.

Variable	1	2	3	4	5	6
1. Engagement	—					
2. Exhaustion	−0.48 ***	—				
3. Cynicism	−0.55 ***	0.78 ***	—			
4. Inadequacy	−0.41 ***	0.64 ***	0.65 ***	—		
5. AtnmSpt	0.55 ***	−0.38 ***	−0.40 ***	−0.29 ***	—	
6. AtnmSpr	−0.34 ***	0.43 ***	0.43 ***	0.32 ***	−0.50 ***	—
M	29.22	6.73	4.91	3.91	29.78	16.28
SD	6.18	2.80	2.16	1.57	5.38	5.06

*Note.* *** *p* < 0.001. AtnmSpt = autonomy support; AtnmSpr = autonomy suppress.

**Table 2:** Fit indices for the compared mixture models.

No. of Profiles	FP	LL	AIC	BIC	SABIC	Entropy	ALMR (*p*)	BLRT (*p*)	Smallest Proportion
1	8	−4013.078	8042.16	8074.33	8048.94	—	—	—	—
2	13	−3745.755	7517.51	7569.78	7528.53	0.826	0.0032	0.0000	38.6%
3	18	−3561.073	7158.15	7230.53	7173.41	0.929	0.0003	0.0000	5.3%
4	23	−3488.809	7023.62	7116.10	7043.12	0.956	0.0030	0.0000	3.6%
5	28	−3441.084	6938.17	7050.76	6961.91	0.930	0.3347	0.0000	3.4%

*Note.* FP = free parameters, LL = log-likelihood value, VLMR (*p*) = *p*-value for VLMR test, BLRT (*p*) = *p*-value for BLRT test.

**Table 3:** Unstandardized final latent profile solution.

	Engagement		Exhaustion		Cynicism		Inadequacy	
	M	SD	M	SD	M	SD	M	SD
Moderately engaged	27.27	5.07	7.53	1.78	5.84	0.72	4.45	1.06
Engaged	32.40	4.61	4.98	1.58	3.17	0.38	3.03	1.30
Moderately burned out	22.05	4.32	10.08	1.96	8.22	0.80	5.31	1.01
Burned out	22.53	10.46	14.60	1.45	11.33	0.82	7.13	1.25

**Table 4:** Estimated Log odds for teaching methods predicting latent profile membership.

	Moderately Engaged vs. *Engaged*	Moderately Engaged vs. *Moderately Burned Out*	Moderately Engaged vs. *Burned Out*	Engaged vs. *ModeratelyBurned Out*	Engaged vs. *Burned Out*	Moderately Burned Out vs. *Burned Out*
	β	β	β	β	β	β
Autonomy support	−0.110 ***	0.109 *	0.025	0.169 ***	0.075	−0.050
Autonomy suppress	0.120 ***	−0.112 *	−0.093	−0.170 ***	−0.131 ***	0.000

*Note.* * *p* < 0.05; *** *p* < 0.001. Groups printed in italics are the reference group.

## Data Availability

The data presented in this study are available on request from the corresponding author.
